# Understanding Recruitment Yield From Social Media Advertisements and Associated Costs of a Telehealth Randomized Controlled Trial: Descriptive Study

**DOI:** 10.2196/41358

**Published:** 2023-05-18

**Authors:** Jéssica Bianca Aily, Jennifer Copson, Dana Voinier, Jason Jakiela, Rana Hinman, Megan Grosch, Colleen Noonan, Megan Armellini, Laura Schmitt, Mika White, Daniel White

**Affiliations:** 1 Department of Physical Therapy University of Delaware Newark, DE United States; 2 Department of Physical Therapy Universidade Federal de São Carlos São Carlos Brazil; 3 Department of Physiotherapy University of Melbourne Melbourne Australia

**Keywords:** remote recruitment, Facebook ads, knee osteoarthritis, consent, screening, social media, telehealth, clinical, recruitment, treatment, osteoarthritis, knee

## Abstract

**Background:**

Recruiting study participants for clinical research is a challenging yet essential task. Social media platforms, such as Facebook, offer the opportunity to recruit participants through paid advertisements. These ad campaigns may be a cost-effective approach to reaching and recruiting participants who meet specific study criteria. However, little is known about the extent to which *clicks* on social media advertisements translate to the actual consent and enrollment of participants who meet the study criteria. Understanding this is especially important for clinical trials conducted remotely, such as telehealth-based studies, which open the possibility to recruit over large geographical areas and are becoming more common for the treatment of chronic health conditions, such as osteoarthritis (OA).

**Objective:**

The aim of this study was to report on the conversion of clicks on a Facebook advertisement campaign to consent to enrollment in an ongoing telehealth physical therapy study for adults with knee OA, and the costs associated with recruitment.

**Methods:**

This was a secondary analysis using data collected over the first 5 months of an ongoing study of adults with knee OA. The Delaware Physical Exercise and Activity for Knee Osteoarthritis program compares a virtually delivered exercise program to a control group receiving web-based resources among adults with knee OA. Advertisement campaigns were configured on Facebook to reach an audience who could be potentially eligible. Clicking on the advertisement directed potential participants to a web-based screening form to answer 6 brief questions related to the study criteria. Next, a research team member called individuals who met the criteria from the screening form and verbally asked additional questions related to the study criteria. Once considered eligible, an electronic informed consent form (ICF) was sent. We described the number of potential study participants who made it through each of these steps and then calculated the cost per participant who signed the ICF.

**Results:**

In sum, between July and November 2021, a total of 33,319 unique users saw at least one advertisement, 9879 clicks were made, 423 web-based screening forms were completed, 132 participants were successfully contacted, 70 were considered eligible, and 32 signed the ICF. Recruitment costed an average of US $51.94 per participant.

**Conclusions:**

While there was a low conversion from clicks to actual consent, 32% (32/100) of the total sample required for the study were expeditiously consented over 5 months with a per-subject cost well below traditional means of recruitment, which ranges from US $90 to US $1000 per participant.

**Trial Registration:**

Clinicaltrails.gov NCT04980300; https://clinicaltrials.gov/ct2/show/NCT04980300

## Introduction

Investigators traditionally recruit participants for clinical research using flyers, print and broadcast media, and word of mouth. Use of web-based recruitment methods have grown over the past 20 years with the inception of social media [1-4]. For example, Facebook is used by approximately 74% of adult American women and 62% of adult American men, and is widely used in all age, racial, and ethnic groups (73% of Hispanic Americans, 70% of Black Americans, and 67% of White Americans) [5]. Moreover, Facebook allows advertisers to target audiences by age, sex, marital status, geographic region, and personal interest characteristics. Hence, it is no surprise that Facebook has become more commonly used in clinical research as a tool to recruit participants in the last decade [1,4-6].

A major gap in planning for web-based physical therapy intervention studies is that little is known about the extent to which reach and clicks on advertisements translate to actual consent and enrollment of participants who meet study criteria, and the costs associated with recruitment. Reach is the number of unique people who have seen a post on Facebook [7]. In addition, it is unclear if reach, clicks, and consent differ by geographic location, age, and sex. Historically, younger adults use social media and web-based platforms. However, recent data show older adults are the most rapidly growing demographic among social media users [8]. Understanding the conversion of reach and clicks to consent, and costs, geographic extension, and differences between age and sex are important for planning future clinical studies, especially those using a remote intervention.

The primary aim of this study was to report on the conversion of reach and clicks from a Facebook advertising campaign to enrollment defined as electronically signing the study informed consent form (ICF) in an ongoing nationwide telehealth physical therapy study for adults with knee osteoarthritis (OA) within the United States. Secondarily, recruitment costs, geographic characteristics, age, and sex were described for reach, clicks, and consent from advertisements.

## Methods

### Study Design and Participants

We use data from on an ongoing study that is remotely recruiting 100 adults with knee OA over 16 months. Our recruitment goal was to ramp up to enrolling 6 adults per month who met the study criteria, starting in the fourth month of the trial. In sum, we had a goal of recruiting 22 study participants within the first 5 months of the study, based on a prior pilot study with the same recruitment method. Briefly, the study randomizes consenting participants into either a brief intervention of web-based resources or expanded intervention of physical therapist–prescribed, virtually delivered exercises with outcome measures taken at baseline, 12 weeks, and 24 weeks. The study criteria require participants to be at least 45 years old, meet a clinical diagnosis of knee OA, [9] and reside within the contiguous United States. A detailed description of the study inclusion and exclusion criteria can be found in Multimedia Appendix 1. Additionally, study participants must pass an adult pre-exercise screening system questionnaire [10], designed to identify individuals who may be at an increased risk of an adverse event during exercise.

### Ethical Considerations

The trial was approved by the institutional review board (IRBNet ID: 1730922-23) at the University of Delaware and is registered at ClinicalTrials.gov (NCT04980300). Participants considered eligible received an electronic ICF, and after agreeing to participate, they signed the ICF. In addition, to guarantee the participants’ confidentiality, neither their name nor any identifying information was used.

The participants received a US $25 Amazon gift card upon completion of the 12-week data collection, and a US $25 Amazon gift card upon completion of the 24-week data collection.

### Recruitment

We recruited participants using paid Facebook advertisements. Facebook uses proprietary algorithms to target and deliver content to specific users by evaluating and scoring advertisements based on the user’s clicks on content within their feed. In addition, Facebook allows basic targeting based on age, gender, and location from an individual’s profile page. Using Facebook’s ability to target demographic groups, we directed our advertisements to men and women of at least 45 years of age, who reside in the contiguous United States (excludes Hawaii and Alaska). Facebook also allows additional ad targeting based on interests and activities. Thus, we set up our campaigns based on the following keywords: knee, knee pain, physical therapy, physical therapist, exercise, osteoarthritis, arthrosis, arthritis, and joint pain. When respondents clicked on the ad, they were directed to a Research Electronic Data Capture (REDCap)–based screening form. REDCap is a secure web application for building and managing databases and is used to collect virtually any type of data.

### Facebook Ad Campaigns

**Figure 1:**
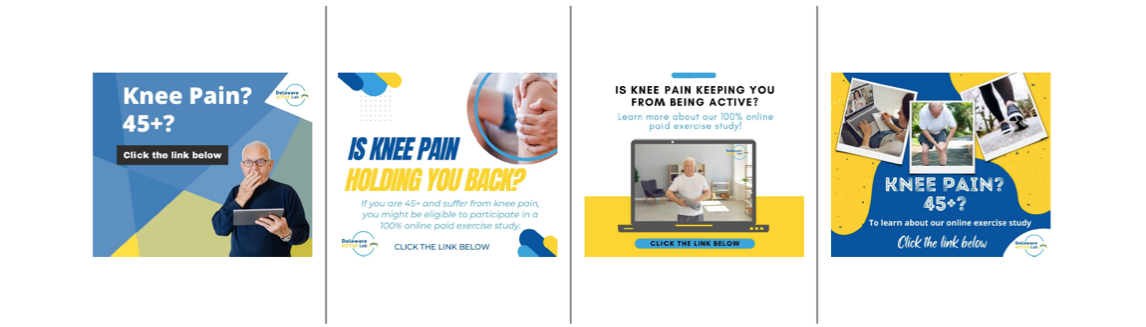
Facebook advertisements.

We created 7 separate advertisement campaigns that ran from July 1 through November 30, 2021. The Facebook ad structure includes basic text next to an image. The advertisements are pictured in Figure 1. All campaigns were available for a minimum period of 2 days and a maximum period of 7 days.

Clicking on the advertisement directed potential participants to a web-based screening form to answer 6 brief questions related to the study criteria, such as age, activity-related knee pain, management of knee OA, and intention to be more active. If the individual provided answers that made them potentially eligible for the study, REDCap asked them to provide their phone number and displayed a message that a research team member would contact them shortly. If ineligible, REDCap automatically displayed a message that they were ineligible and thanked them for their interest.

Lastly, a research team member called individuals who met the criteria from the screening form to confirm their answers within 7 days and then asked additional questions to ensure they met the study inclusion and exclusion parameters. These included the following: a clinical diagnosis of knee OA [9] (ie, aged >45 years); activity-related knee pain and morning stiffness <30 min per day; limited regular exercise <60 min per week; no scheduled total knee replacement; and no physical therapy or formal strength training program for the lower limbs within the past 6 months. Once considered eligible, an electronic ICF was emailed to the participant.

### Study Outcome Measures

#### Recruitment Metrics

We assessed eligibility and enrollment over 5 months. Metrics related to Facebook advertisements were tracked through the Facebook Analytics Tool, which recorded reach (the number of unique people who have seen a post on Facebook) and clicks to the advertisements. We calculated the overall number of reaches and clicks as well as within age groups and sex categories.

#### Study Enrollment

We defined a “being screened” participant as a study participant who completed the screening form. We defined a participant as “eligible” if they met all the study inclusion and exclusion criteria. We defined “consenting to participate” as participants who signed the ICF and returned it to us. The numbers of participants who were screened, eligible, and consented to participate in the study were counted.

#### Geographical Representation

We defined geographical representation by the number of contiguous states in which participants who signed the ICF resided.

#### Recruitment Costs

Recruitment cost was calculated as the amount spent during the Facebook advertisement campaigns, which is charged by the number of clicks. We calculated the per-participant cost by dividing the total advertising costs by the number of participants who signed the ICF.

### Statistical Analysis

Descriptive statistics for continuous measures were provided using mean and standard deviation. Counts and percentages were provided for categorical variables. We describe reach, clicks, and consent stratified by sex (female and male) and age (45 to 54 years; 55 to 64 years; and older than 65 years).

## Results

**Figure 2:**
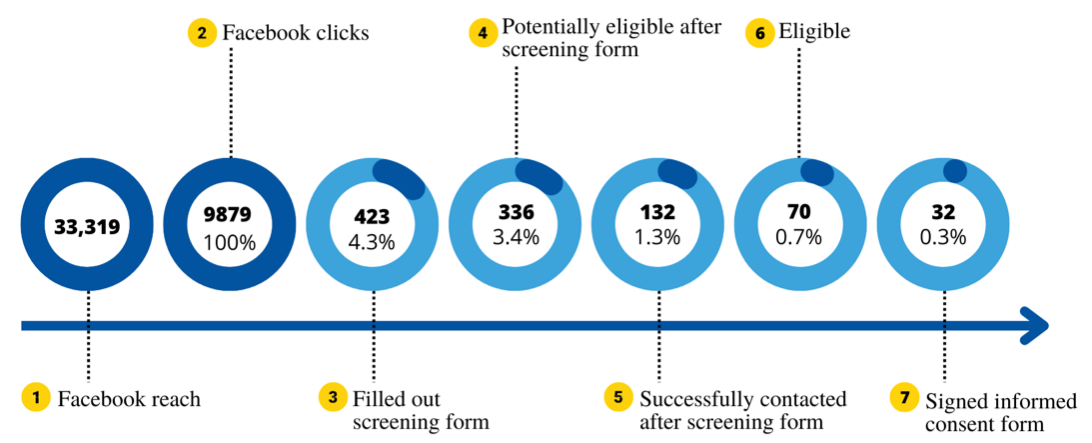
Timeline between Facebook reach and clicks to consent.

A total of 7 advertisement campaigns were conducted on Facebook from July to November 2021, each lasting an average of 4 days. In sum, 33,319 unique users across the 48 states in the contiguous United States saw at least one advertisement (reach). A total of 9879 clicks were made, and 423 (4.3%) screening forms were completed. Of those 9879 clicks, 336 (3.4%) participants passed the screening form criteria and 132 (1.3%) were successfully contacted by a research assistant (n=11, 0.1% did not provide contact information, and n=193, 1.96% could not be reached). Of the 9879 persons who clicked the advertisements, 70 (0.7%) were considered eligible, and 32 participants (0.3%) signed the ICF, which exceeded our enrollment target of 22 study participants within the first 5 study months. In particular, the ICF was signed by 12, 3, 4, 7, and 6 adults in the first 5 study months, respectively. This sample of 32 participants included 24 (75%) women, and the average age was 59.8 (SD 8.4) years (Figure 2).

**Figure 3:**
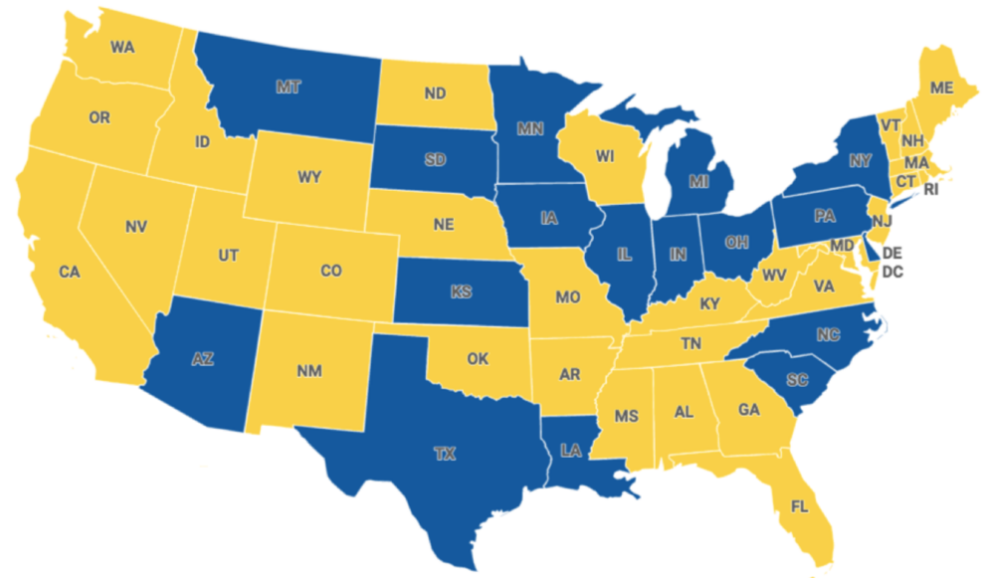
The US geographical representation.

Of the 9879 clicks on the study advertisement, 32 (0.3%) were ultimately eligible. Campaigns cost a total of US $1661.97, and cost per consented participant was US $51.94. The 32 participants who were enrolled came from 17 (35.4%) of the possible 48 different states. In addition, it is important to note that people from all contiguous United States (48 states) were reached (Figure 3).

**Figure 4:**
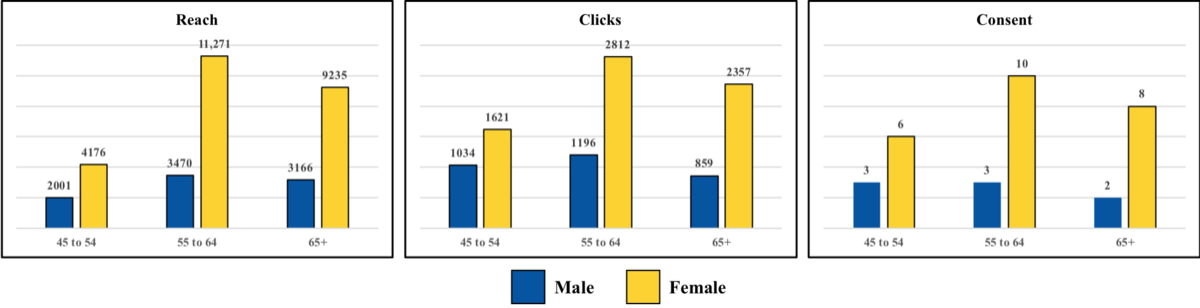
Facebook metrics by age groups and sex.

Participants aged between 55 and 64 years had the highest reach, clicks, and consents followed by those aged >65, while those 45 to 54 years old had the lowest reach, clicks, and consent. In terms of sex, women had higher numbers of reach, clicks, and consents compared to men (Figure 4).

## Discussion

### Principal Findings

We reached over 30,000 Facebook users, and close to 10,000 adults clicked on our Facebook advertisement. From these clicks, we enrolled 32% (32/100) of our required study sample in only 5 months, and spent US $51.94 per participant who ultimately signed the ICF. This sample came from 17 different states, with ages ranging from 46 to 77 years (mean 59.8 years), with more women (n=24, 75%) than men (n=8, 25%), and more White (n=28, 88%) than Black or African Americans (n=3, 9%) and Asians (n=1, 3%). These findings point to social media being a useful and efficient avenue to recruit and enroll participants with knee OA within the United States into a telehealth physical therapy study.

We observed a steep drop-off from clicks to actual enrollment. In other words, a large portion of participants did not fill out the screening form after being redirected to the survey link as we had 9879 clicks, but only 423 (4.3%) filled out the screening form. Although speculative, these participants may have clicked on the link without carefully reading the advertisements, or they may have lost interest after reading the full study description. Developing credibility and trust with an investigative team is an important element for clinical trials, and the absence of additional channels (eg, email and phone number) to contact the research team may have eroded the participant’s confidence and trust with the integrity of the study [11]. Furthermore, less than half of those participants considered eligible (n=70) signed the ICF.

Similar drop-off was reported by Reagan et al [6] in a review of studies that recruited participants through Facebook, which found a range of 388,630 reaches resulting in 259 clicks among the included studies; however, the actual participant enrollment yield was even smaller, approximately 19.2% (range: 2.5%-80%) [6]. Additionally, it is important to note that the same drop-off occurs using traditional means of recruitment through printed media, such as newspapers, magazines, and flyers, as well as community events. In a previous randomized controlled trial investigating standard care versus celecoxib, a newspaper advertising campaign with a daily circulation of over 570,000 people conducted over 8 weeks yielded 320 phone calls, but only 15 volunteers met the study criteria [12]. Thus, the low relative yield from clicks to consent found on the web is not unique, but rather common to participant recruitment into clinical trials in general.

Our recruitment costs were far less than traditional methods. Our average recruitment cost of US $51.94 per participant is well below figures presented by Tate et al [13] of US $1094.27 per participant for television advertising, US $811.99 for printed media (local newspapers, magazines, and campus newspaper), US $635.92 for radio, US $96.71 for website recruitment, and US $96.90 for flyers and community events when recruiting for a study on the prevention of weight gain in young adults [13,14]. The low cost of recruiting participants further supports the feasibility and advantage of using social media to recruit participants [14,15].

A notable aspect of our sample was that it represented 17 different states within the United States, with approximately one-third coming from predominantly rural-area states. Correspondingly, a recent US-based study that included remotely delivered interventions found that virtually recruited strategies yielded a larger number of states (average of 24.4 states) compared to traditionally recruited strategies (average of 1.1 states) [16]. Thus, web-based recruitment may help facilitate recruitment from a wider geographic area than studies using traditional means of recruitment.

We find it notable that our average age was 59.8 years, with most participants (41%, n=13) falling into the 55-64 years age category. Moreover, we recruited more women than men, and the average age of our sample was 59.8 (SD 8.4) years. These findings are consistent with a previous unpublished study, which successfully recruited adults older than 40 years with OA from Facebook for a clinical trial [17]. Older adults’ adoption of the internet is steadily increasing with a recent Pew Research Center study indicating that 77% of those aged 30 to 49 use Facebook, followed by 73% of those aged 50 to 64 [5]. The recruitment of predominantly women was somewhat expected given that there are more women Facebook users than men (74% of US women vs 62% of US men) [5]. This is relevant to our study since knee OA is more common in women than men [18,19] and it is thus appropriate to recruit a sample more biased toward women than men.

### Limitations

Our study had a few limitations. First, this study uses data collected as part of the recruitment processes for an ongoing trial. Thus, there is a possibility that the estimates of reach, clicks, and consent may change when our study is completed, although this is unlikely since our findings are consistent with previously published studies. Additionally, although there is an assumption that patients consider remote interventions more favorably as a matter of convenience, we did not access participants’ preference to either telehealth or face-to-face trials. Second, it is not known how generalizable our study findings are to geographical areas outside the United States or studies that are more controlled or have more stringent exclusion criteria. Finally, we did not aim to compare Facebook recruitment with other social media, such as Instagram and Twitter, or with traditional recruitment methods (eg, flyers, print and broadcast media, and word of mouth) since this was beyond the scope of our study.

### Conclusion

Advertising on Facebook offers the opportunity to recruit potential study participants nationwide for a web-based physical therapy telehealth study at less cost per person than traditional recruitment methods. Out study adds to the previous literature that few people who clicked on advertisements were ultimately consented into our study. Nevertheless, we still recruited 32% of our total target sample over the first 5 months of our study. In addition, women and individuals aged between 55 and 64 years are the most reached by Facebook advertisements, and consequently, are the people who clicked and consented to participate in our study more frequently. In all, it is recommended that future researchers consider using social media for recruiting adults with knee OA for clinical research given that we found Facebook to be a feasible, cost-saving, and efficient method to recruit study participants for a nationwide telehealth physical therapy study within the United States.

## Appendix

Multimedia Appendix 1 Inclusion and exclusion criteria of the Delaware Physical Exercise and Activity for Knee Osteoarthritis study.

## Abbreviations

ICF: informed consent form
OA: osteoarthritis
REDCap: Research Electronic Data Capture

## Data Availability

The data sets generated and analyzed during this study are available from the corresponding author on reasonable request.
