# Supplementary material for: Understanding Recruitment Yield From Social Media Advertisements and Associated Costs of a Telehealth Randomized Controlled Trial: Descriptive Study
Source: J Med Internet Res. 2023 May 18;25:e41358. doi: 10.2196/41358 (PMC10236273; doi:10.2196/41358)
Supplement: Multimedia Appendix 1 [file jmir_v25i1e41358_app1.docx]

**SUPPLEMENTARY MATERIAL**

Supplemental Table 1. Inclusion/Exclusion criteria of the Delaware PEAK study

| *Individuals will be included if they:* | *Individuals will be excluded if they:* |
| --- | --- |
| Meet the National Institute for Health and Care Excellence Osteoarthritis (NICE) clinical criteria^5^: | Regularly exercise for more than 60 minutes/week; |
| a. are at least 45 years of age; | Have a scheduled knee replacement; |
| b. have activity-related joint pain that hasn’t already been addressed by knee replacement; | Have had physical therapy, or other exercise program prescribed by a health professional, in the past 6 months; |
| c. have no morning stiffness, or morning stiffness lasting ≤30 minutes; | Have been advised by a physician to not participate in moderate-intensity exercise; |
| Reside in the contiguous United States (i.e., excluding Alaska and Hawaii); |  |
| Comfortable participating in a program delivered in English; |  |
| Have a history of knee pain for at least 3 months; |  |
| Are looking to move more; |  |
| Have either a smartphone or laptop/desktop computer with broadband internet connection; |  |
| Are available for the duration of the intervention portion of the study large trial (12 weeks) and are willing to wear physical activity monitors. |  |
